# Publisher Correction: Evolution of a guarded decoy protease and its receptor in solanaceous plants

**DOI:** 10.1038/s41467-024-46611-2

**Published:** 2024-03-13

**Authors:** Jiorgos Kourelis, Shivani Malik, Oliver Mattinson, Sonja Krauter, Parvinderdeep S. Kahlon, Judith K. Paulus, Renier A. L. van der Hoorn

**Affiliations:** Plant Chemetics Laboratory, Department of Plant Sciences, University of Oxford, South Parks Road, OX1 3RB Oxford, UK

Correction to: *Nature Communications* 10.1038/s41467-020-18069-5, published online 2 September 2020

The original version of this Article contained an error in Fig. 5a, in which the labels E64, Avr2 and Avr2Δ6 were incorrectly aligned with the first, second/third and fourth lanes of the gel respectively, rather than the second, third and fourth lanes as intended. This has been corrected in both the PDF and HTML versions of the Article.

