# Supplementary material for: Evolution of a guarded decoy protease and its receptor in solanaceous plants
Source: Nat Commun. 2020 Sep 2;11:4393. doi: 10.1038/s41467-020-18069-5 (PMC7468133; doi:10.1038/s41467-020-18069-5)
Supplement: Supplementary file 1 — Supplementary Figures [file 41467_2020_18069_MOESM1_ESM.pdf]

Supplementary Figures S1-S8

Evolution of a guarded decoy protease and its receptor in solanaceous plants

Jiorgos Kourelis[1], Shivani Malik[1], Oliver Mattinson[1], Sonja Krauter[1], Parvinderdeep S. Kahlon[1], Judith K. Paulus[1], and Renier A. L. van der Hoorn[1*]

[1] Plant Chemetics Laboratory, Department of Plant Sciences, University of Oxford, South Parks Road, OX1 3RB Oxford, UK

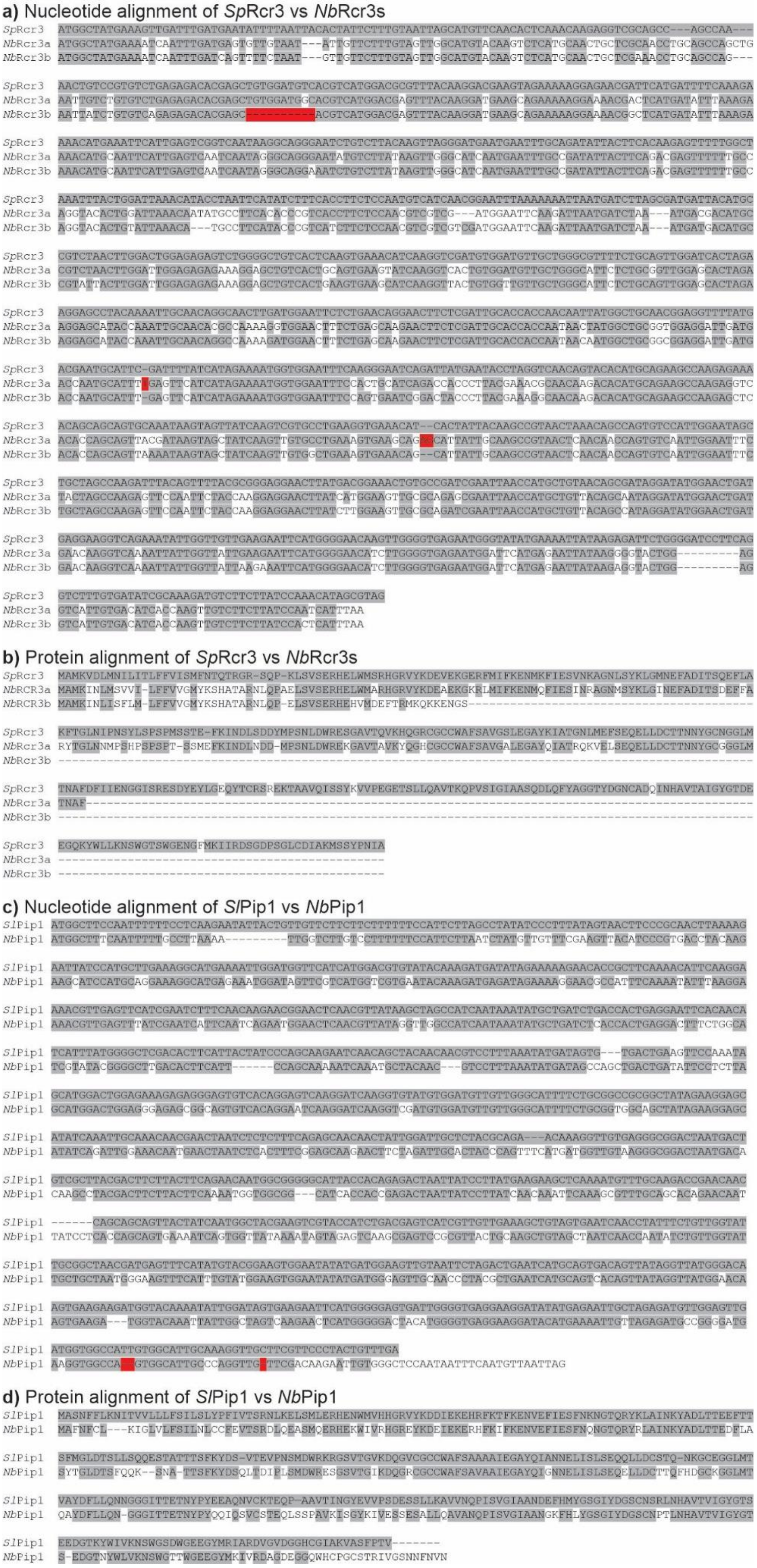

Supplementary Figure 1 Nucleotide and protein alignments of *Nb*Rcr3s and *Nb*Pip1. Sequences were aligned using Clustral Omega. Frameshift mutations are highlighted (red). Used accession codes are: AF493232 (*Sp*Rcr3); Niben101Scf08921-g77214 (*Nb*Rcr3a); Niben101Scf03309-g49741 (*Nb*Rcr3b) NM_001247020.2 (*Sl*Pip1); and Niben101Scf08921-g77212 (*Nb*Pip1).

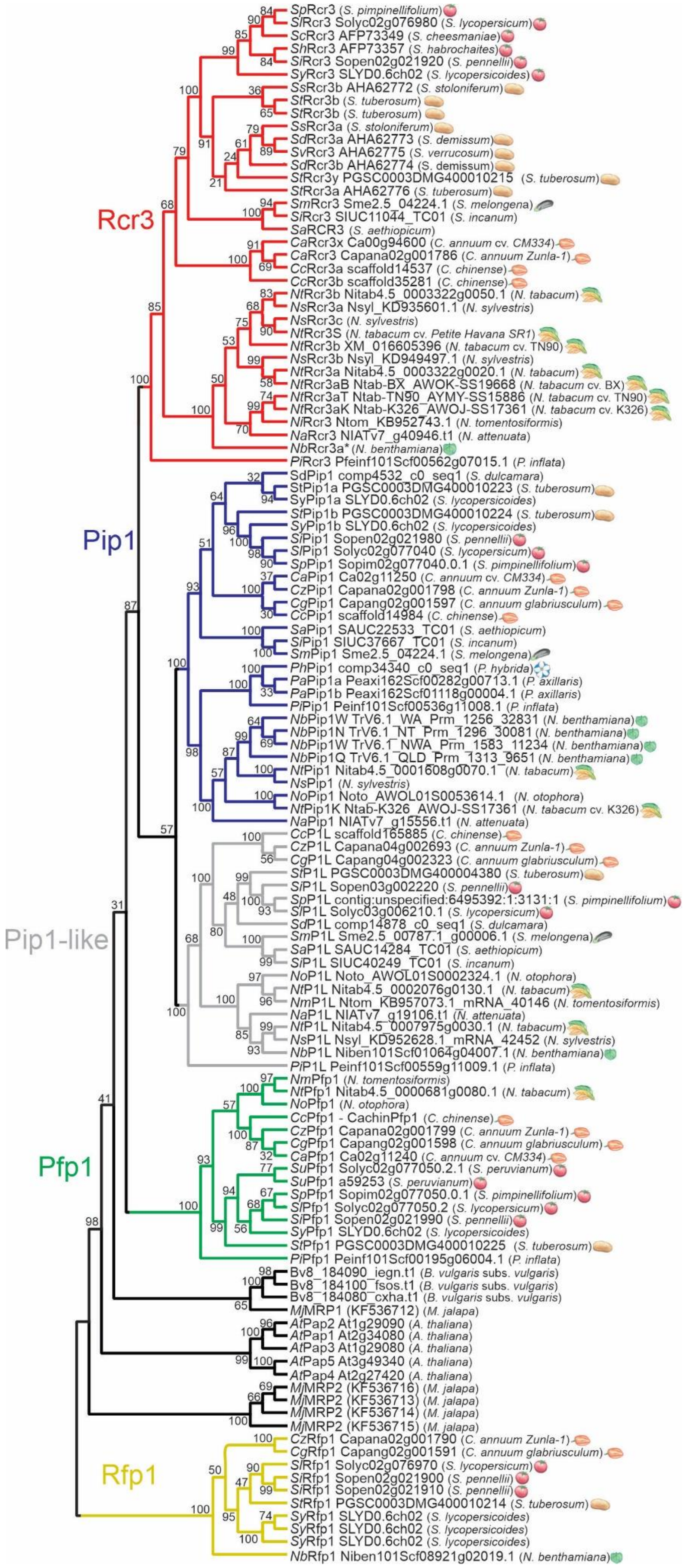

Supplementary Figure 2 Phylogeny of Pip1, Pfp1, Rcr3, Pip1-like PLCPs. The evolutionary history of the Rcr3 clade of proteases was inferred using the Maximum Likelihood method based on the Whelan and Goldman model. The bootstrap consensus tree inferred from 1000 replicates is taken to represent the evolutionary history of the taxa analysed. Shown are percentage bootstrap values.

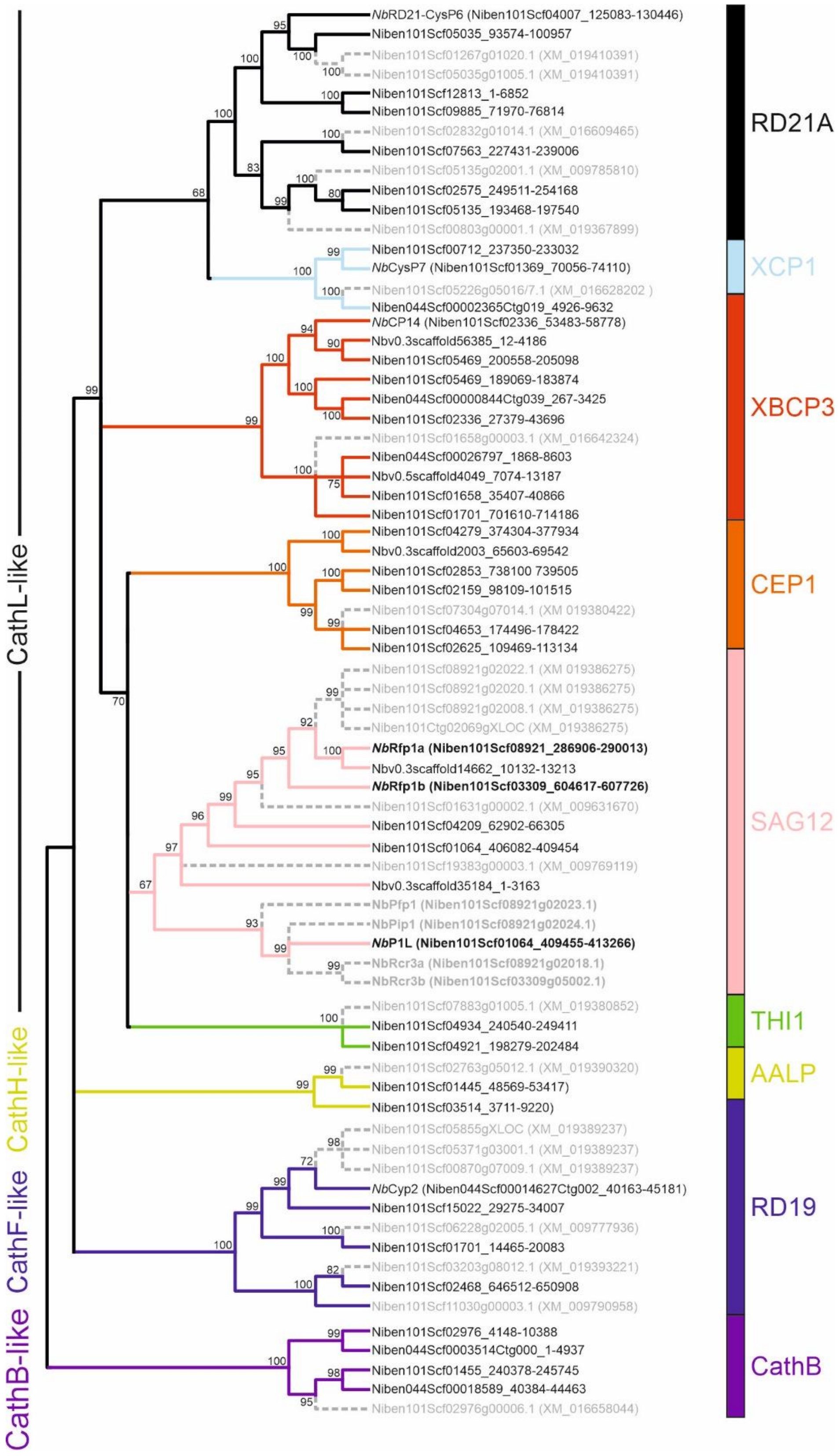

Supplementary Figure 3 Phylogeny of *N. benthamiana* PLCPs. The evolutionary history of the PLCP family was inferred using the Maximum Likelihood method based on the Whelan and Goldman model. The bootstrap consensus tree inferred from 1000 replicates is taken to represent the evolutionary history of the taxa analysed. Shown are percentage bootstrap values. Putative pseudogenes are indicated in grey. Naming of PLCP clades according to (Richau *et al.*, 2012).

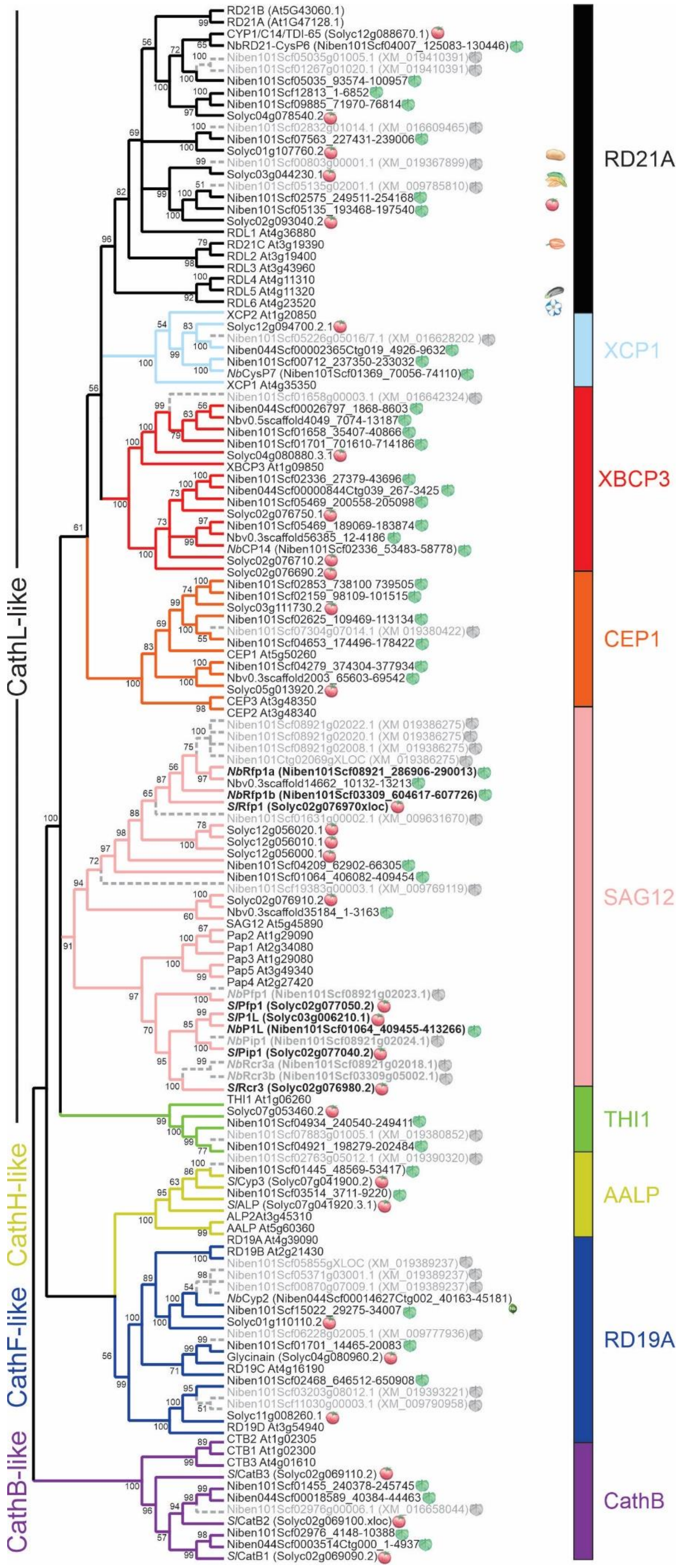

Supplementary Figure 4 Phylogeny of tomato, Arabidopsis, and *N. benthamiana* PLCPs.

The evolutionary history of the PLCP family was inferred using the Maximum Likelihood method based on the Whelan and Goldman model. The bootstrap consensus tree inferred from 1000 replicates is taken to represent the evolutionary history of the taxa analysed. Shown are percentage bootstrap values. Putative pseudogenes are indicated in grey. Naming of PLCP clades according to (Richau *et al.*, 2012).

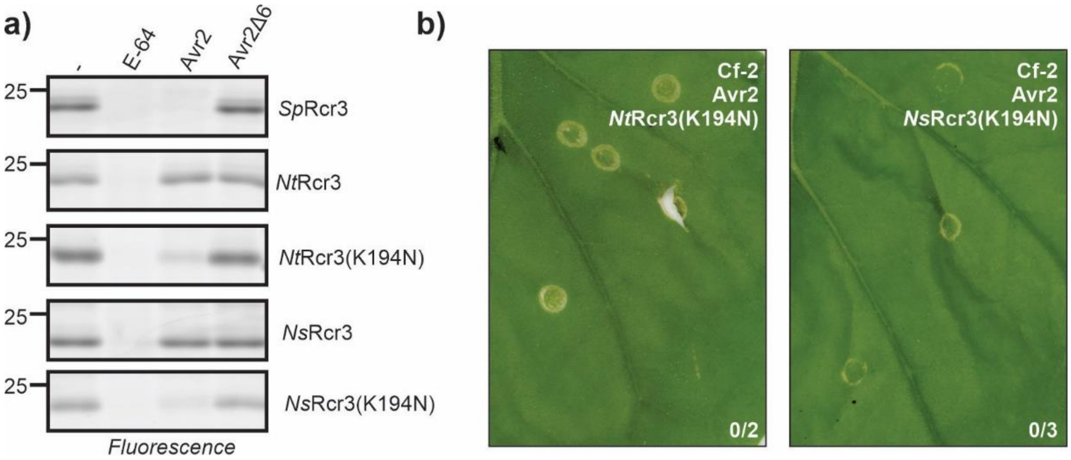

Supplementary Figure 5 *Nicotiana* Rcr3 K194N mutants can be inhibited by Avr2, but do not trigger HR. **a)** Activity profiling of *Sp*Rcr3, and the *Nicotiana tabacum* and *Nicotiana sylvestris* Rcr3 homologs (*Nt*Rcr3 and *Ns*Rcr3, respectively) and the K194N mutants thereof. These proteins were co-expressed with silencing inhibitor P19 in *N. benthamiana* leaves by agroinfiltration. Apoplastic fluids were isolated at 4 dpi, pre-incubated for 45 min with or without 100 µM E-64, 2 µM Avr2 or Avr2Δ6, or DMSO, and labelled for 3 hours with 2 µM MV201. Samples were separated on SDS-PAGE gels and scanned for fluorescence. **b)** Co-expression of Cf-2, Avr2 and the *Nt*Rcr3(K194N) or *Ns*Rcr3(K194N) mutant protease in *N. benthamiana* by agroinfiltration. Binary vectors contained the silencing inhibitor P19 in the backbone, and the Agrobacterium cultures were diluted to OD_{600} = 0.25 each in 1:1:1 mix. Pictures taken at 5 dpi. Numbers indicate the number of leaves showing HR.

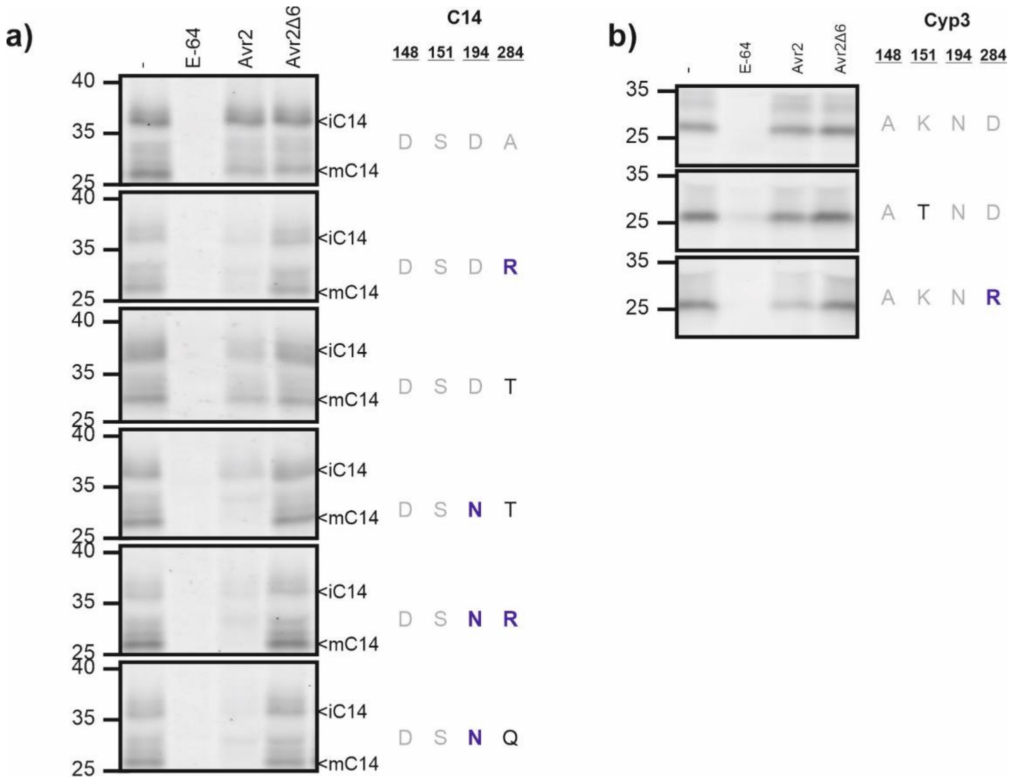

Supplementary Figure 6 Mutating C14 and Cyp3 results in Avr2 inhibition.

a) C14 and, **b)** Cyp3 mutants were co-expressed with the silencing inhibitor P19 by agroinfiltration of *N. benthamiana* leaves. Apoplastic fluids were isolated at 2dpi, pre-incubated for 45min with or without 100µM E-64, 2µM Avr2 or Avr2Δ6, or DMSO, and labelled for 3 hours with 2µM MV201. Samples were separated on SDS-PAGE gels and scanned for fluorescence. The granulin-domain and proline-rich region are removed from active C14 generating an intermediate C14 (iC14) and mature C14 (mC14), respectively (Gu *et al.*, 2012).

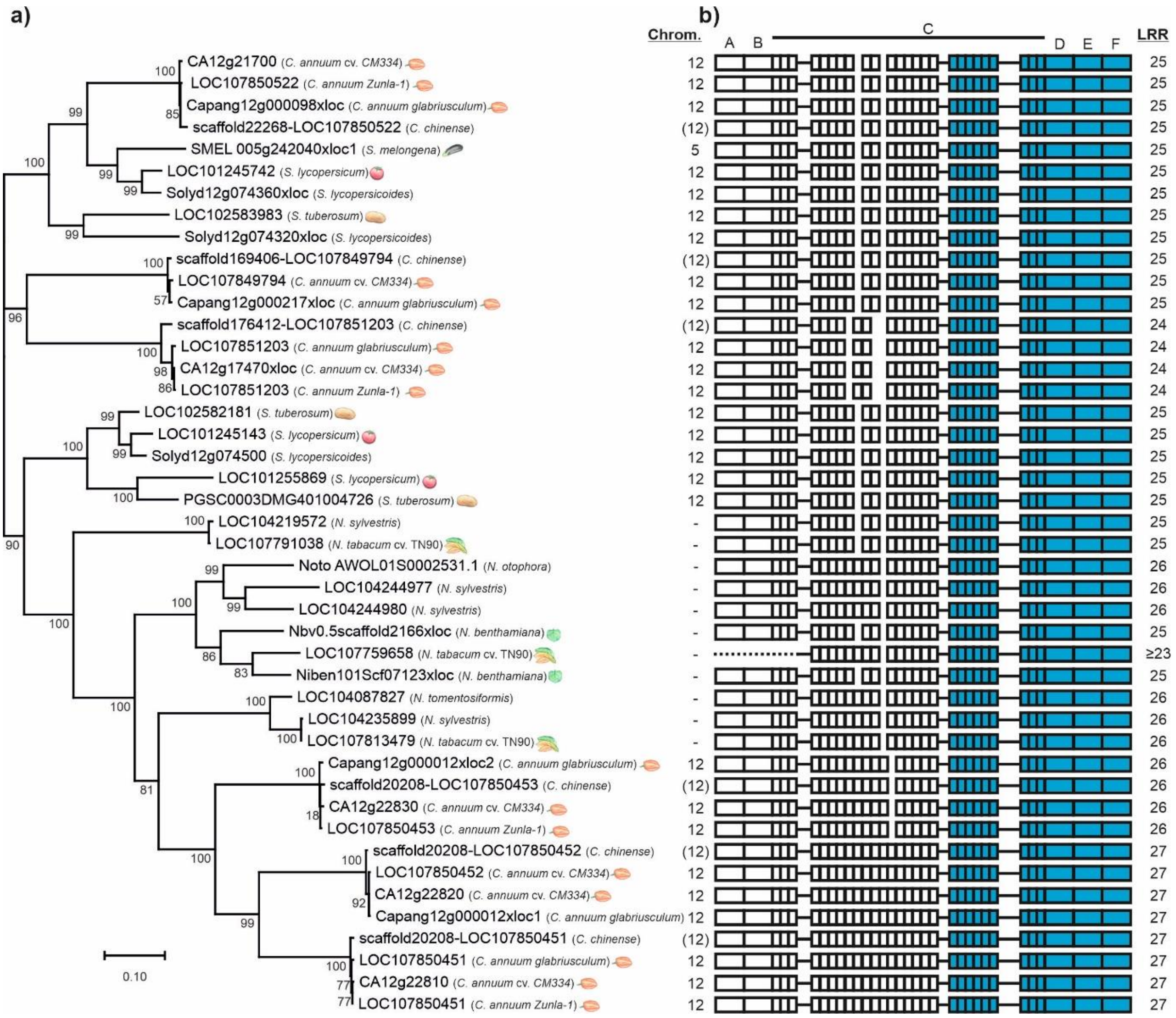

Supplementary Figure 7 The C-terminal half of Cf-2 has been repurposed in another RLP family.

a) Phylogenetic tree of RLPs containing C-terminal homology to Cf-2. Approximate Maximum Likelihood tree was generated using FastTreeMP (Price et al., 2010), based on the Le Gascuel model (Le and Gascuel, 2008). Shown are percentage bootstrap values. The chromosome on which these genes reside is indicated, expected chromosome numbers are indicated in parentheses. Scale bar indicates amino acid substitutions per site. **b)** Amino acid alignment using MUSCLE. LRRs of domain C were predicted using LRRfinder (Offord et al., 2010). Numbers indicate total number of LRRs in domain C. Shown are LRR and C-terminal region with homology to Cf-2 (grey); unknown sequences (dotted lines). A, signal peptide; B, N-terminal LRR flanking domain; C, LRR region; D, juxtamembrane region; E, transmembrane domain; F, cytoplasmic tail.

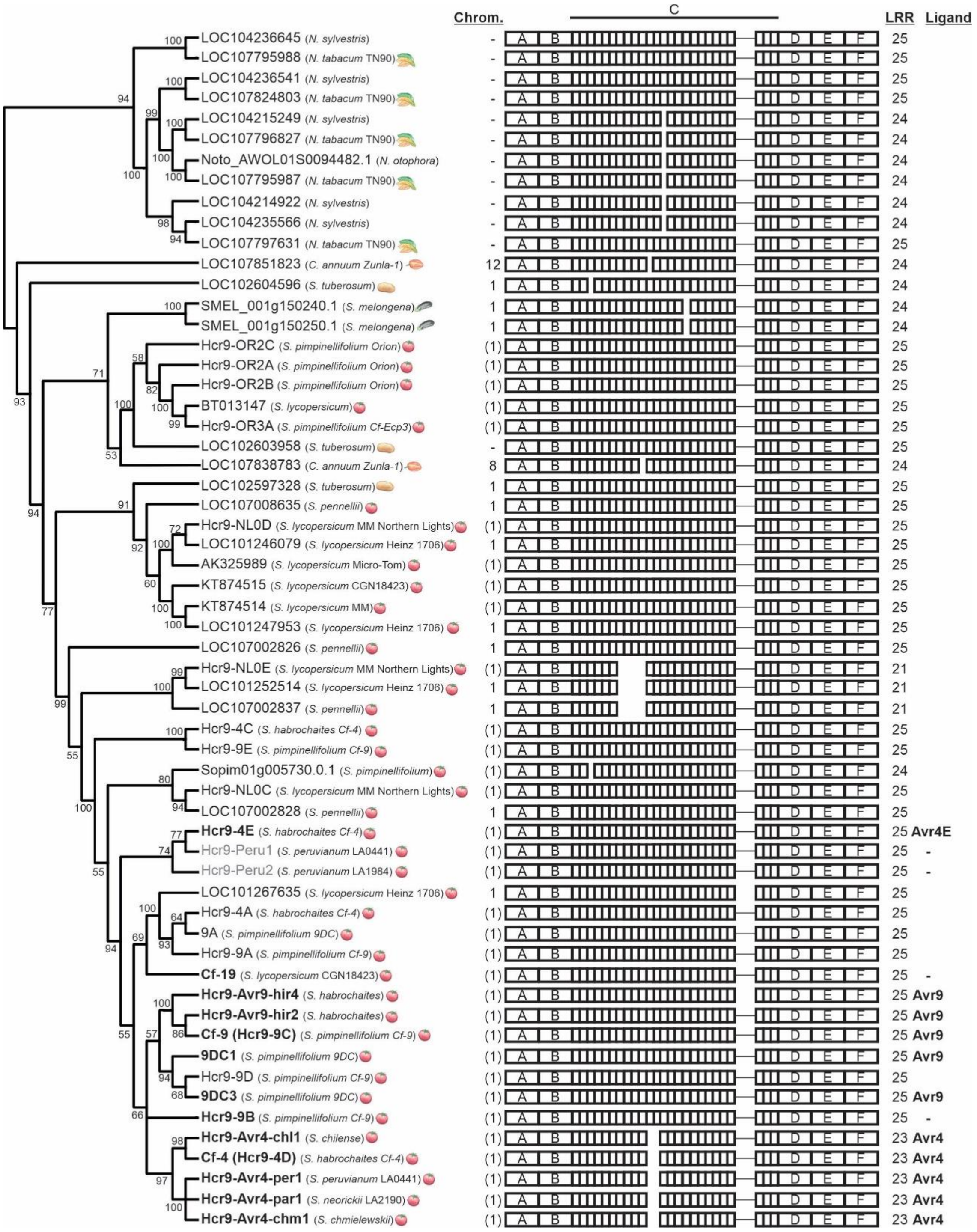

Supplementary Figure 8 Hcr9s are conserved in Solanaceae.

a) Phylogenetic tree of the Hcr9 protein family. The evolutionary history was inferred by using the Maximum Likelihood method based on the based on the Whelan and Goldman model. The bootstrap consensus tree inferred from 1000 replicates is taken to represent the evolutionary history of the taxa analysed. Shown are percentage bootstrap values. Hcr9 family members with known recognition specificity are indicated in bold, in light grey those that induce autonecrosis upon agroinfiltration in *N. benthamiana*, the chromosome on which these genes reside is indicated, expected chromosome numbers are indicated in parentheses. **b)** Alignment of LRRs of domain C to the LRRs of Cf-9 using Clustal Omega. LRRs were predicted using LRRfinder. Numbers indicate total number of LRRs in domain C, putative ligand is indicated for *Hcr9* family members with identified recognition specificities. A, signal peptide; B, N-terminal LRR flanking domain; C, LRR region; D, juxtamembrane region; E, transmembrane domain; F, cytoplasmic tail.
