# Supplementary material for: Evolution of a guarded decoy protease and its receptor in solanaceous plants
Source: Nat Commun. 2020 Sep 2;11:4393. doi: 10.1038/s41467-020-18069-5 (PMC7468133; doi:10.1038/s41467-020-18069-5)
Supplement: Supplementary file 3 — Descriptions of Additional Supplementary Files [file 41467_2020_18069_MOESM3_ESM.pdf]

Descriptions of Additional Supplementary Files

Supplementary Data 1

Description: Genes encoding papain-like Cys proteases from N. benthamiana.

Supplementary Data 2

Description: Solanaceous genes encoding Rcr3- and Pip1-like proteases.

Supplementary Data 3

Description: Used plasmids, with sequences.
