# Supplementary figures and images for: Evolution of a guarded decoy protease and its receptor in solanaceous plants
Source: Nat Commun. 2020 Sep 2;11:4393. doi: 10.1038/s41467-020-18069-5 (PMC7468133; doi:10.1038/s41467-020-18069-5)

Figure 2c

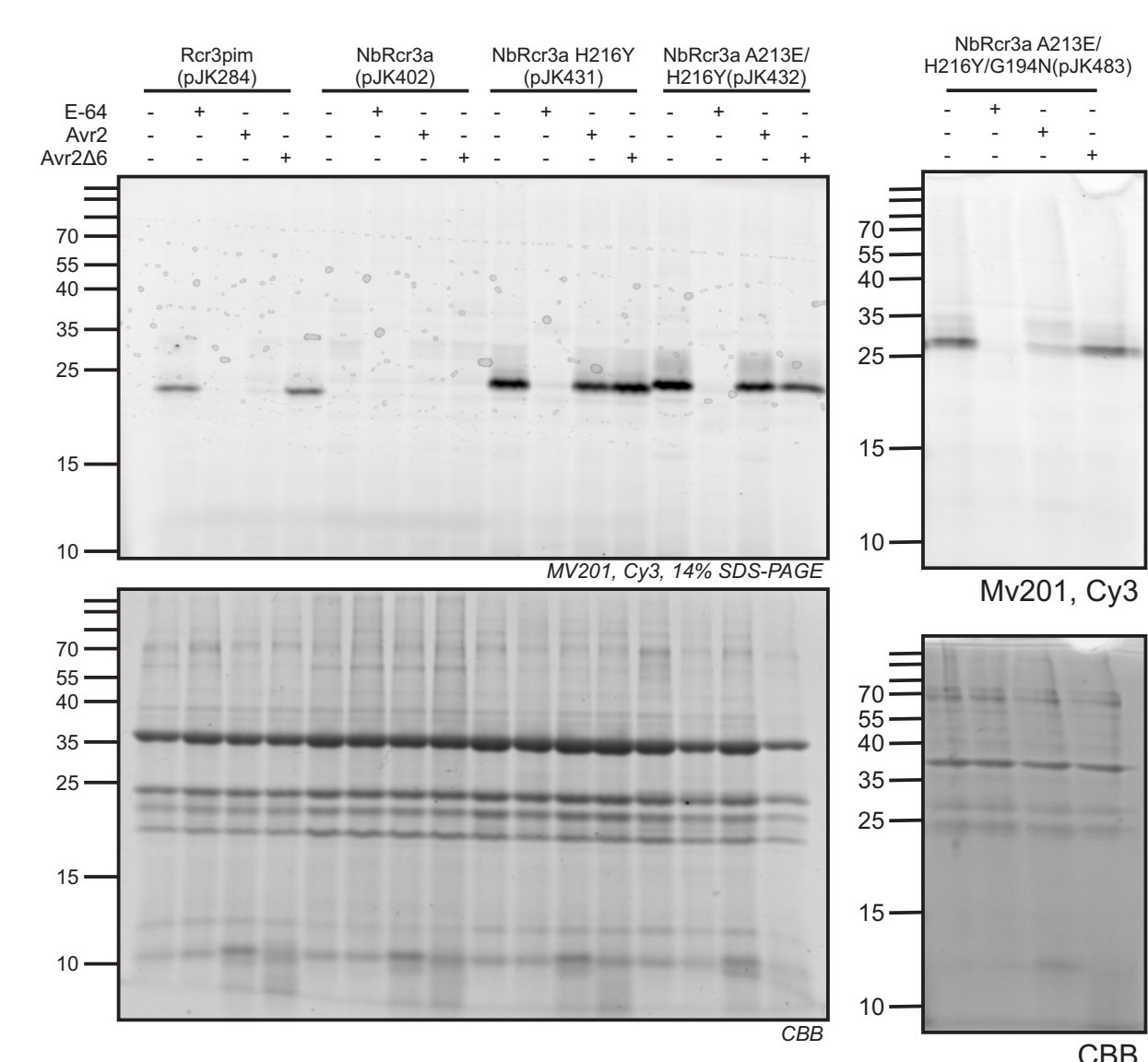

Figure 3f

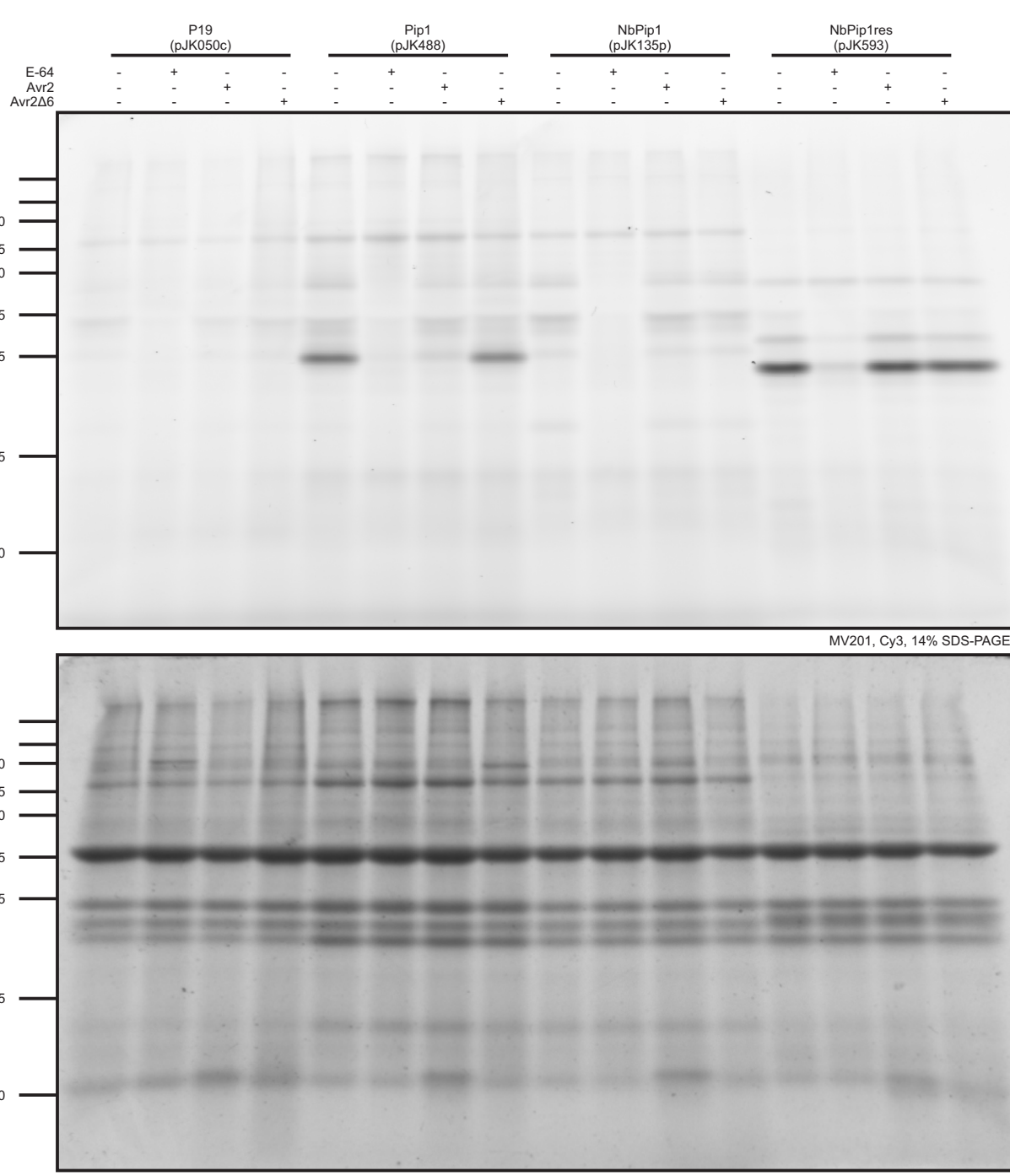

Figure 5a

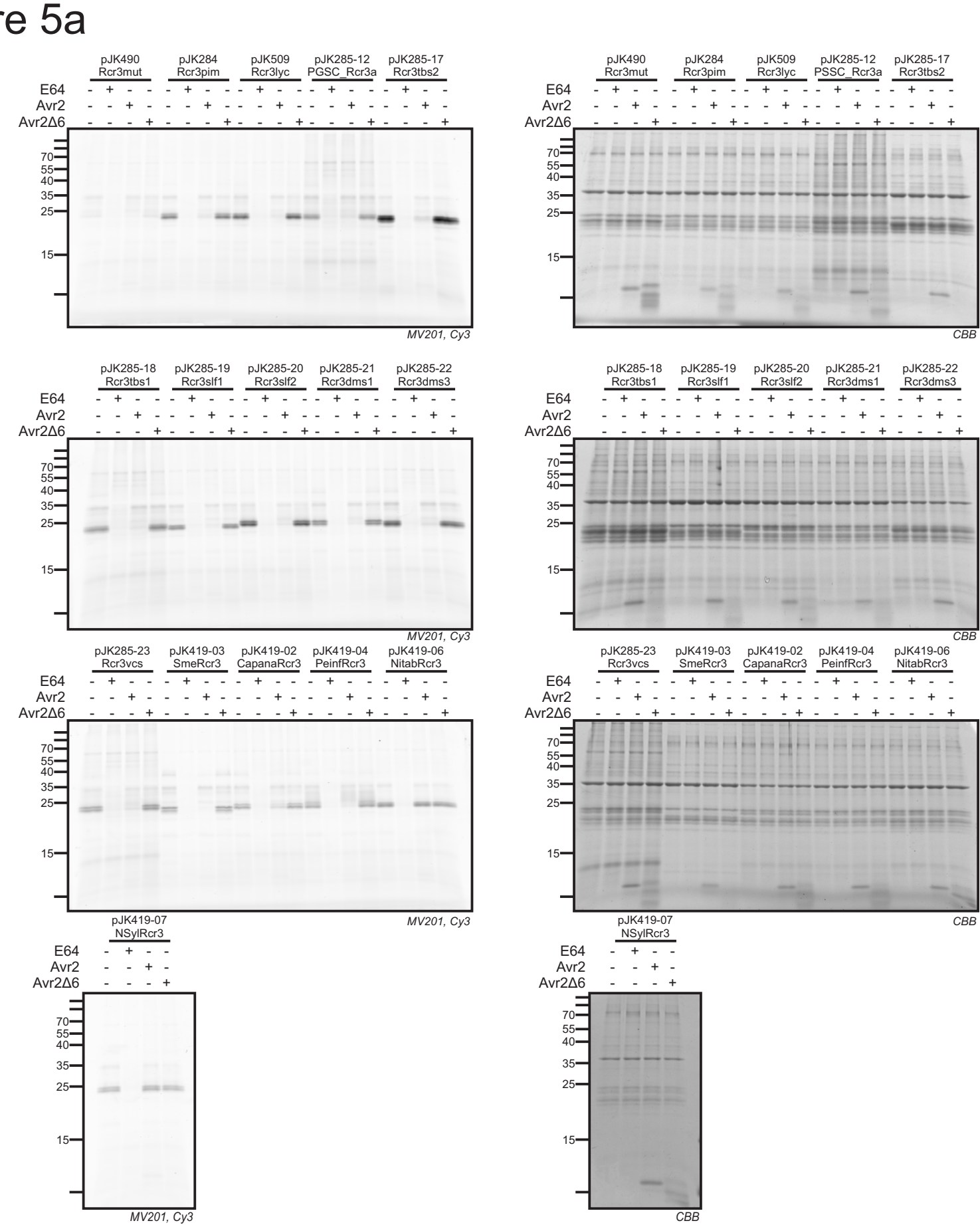

Figure 6b

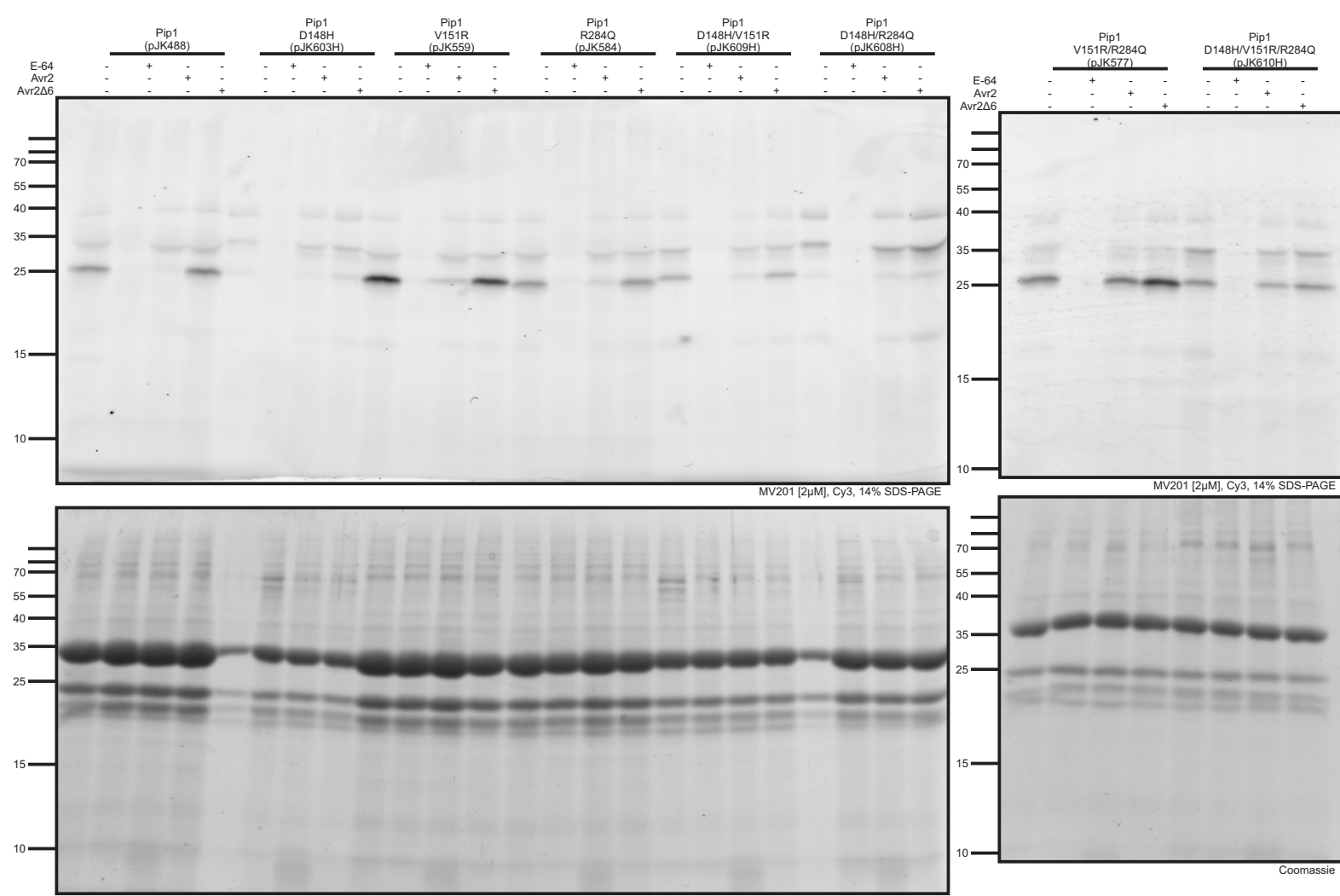

Figure 6a

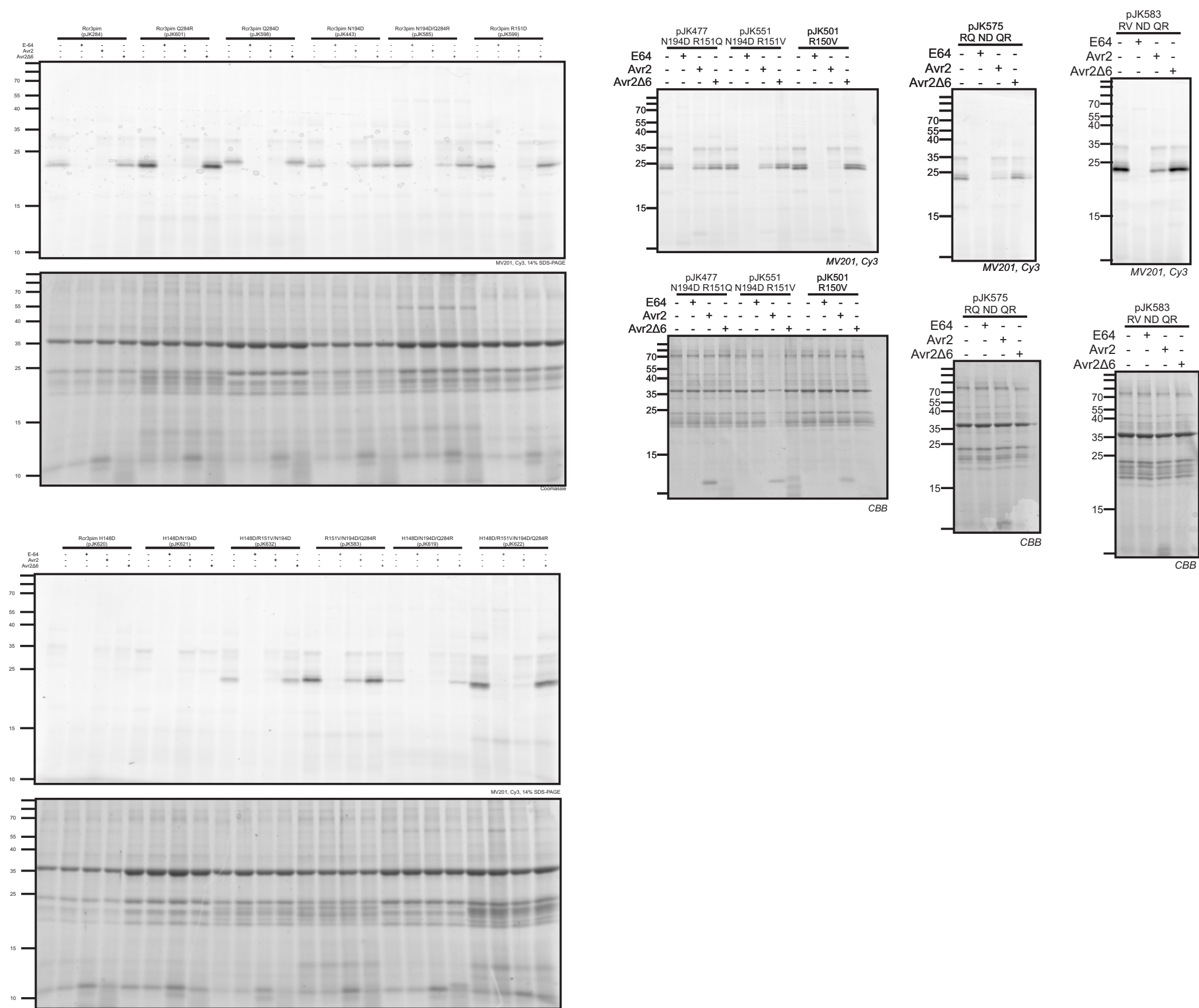

Figure 6f

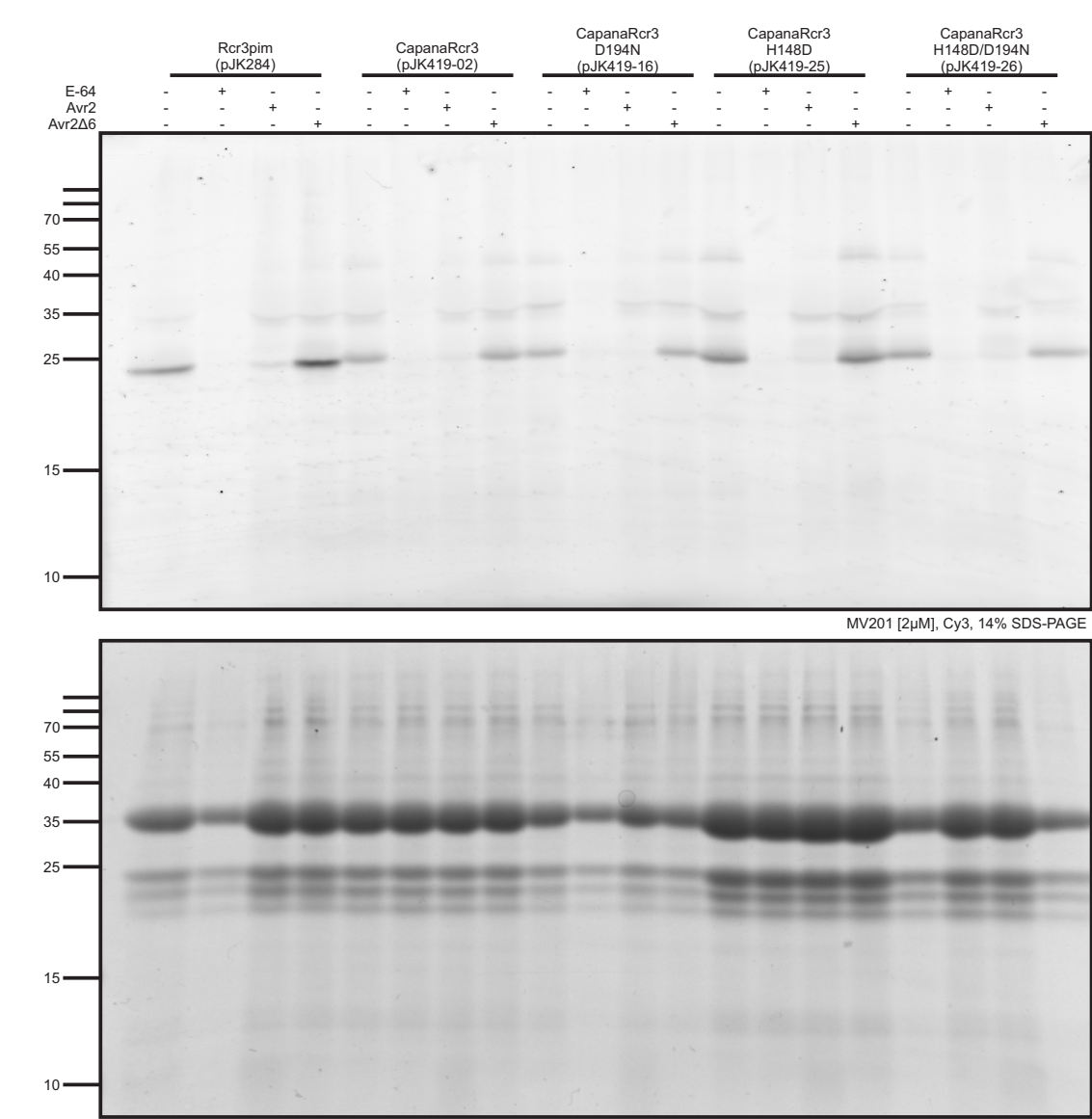

Figure S5

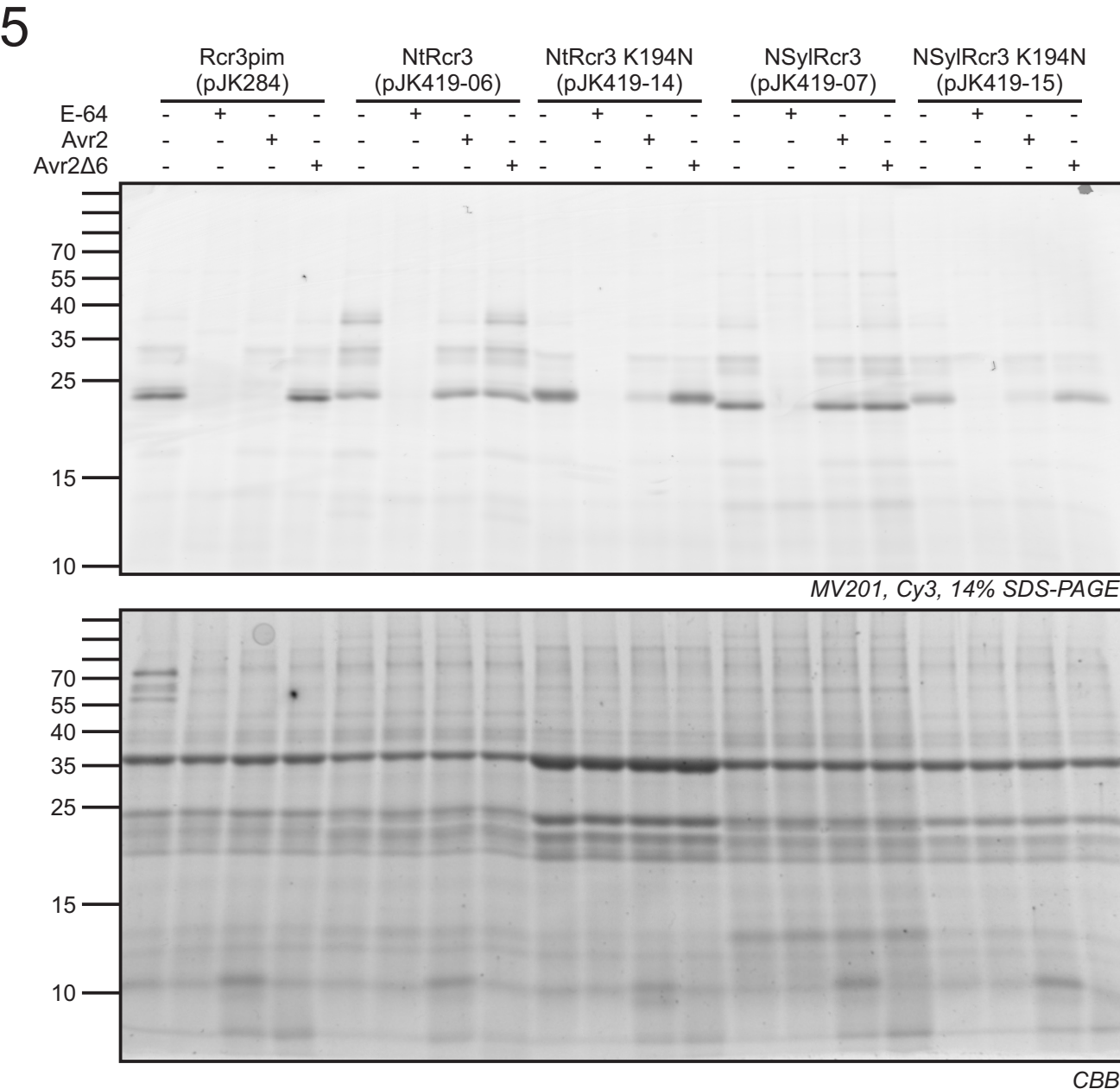

Figure 6c

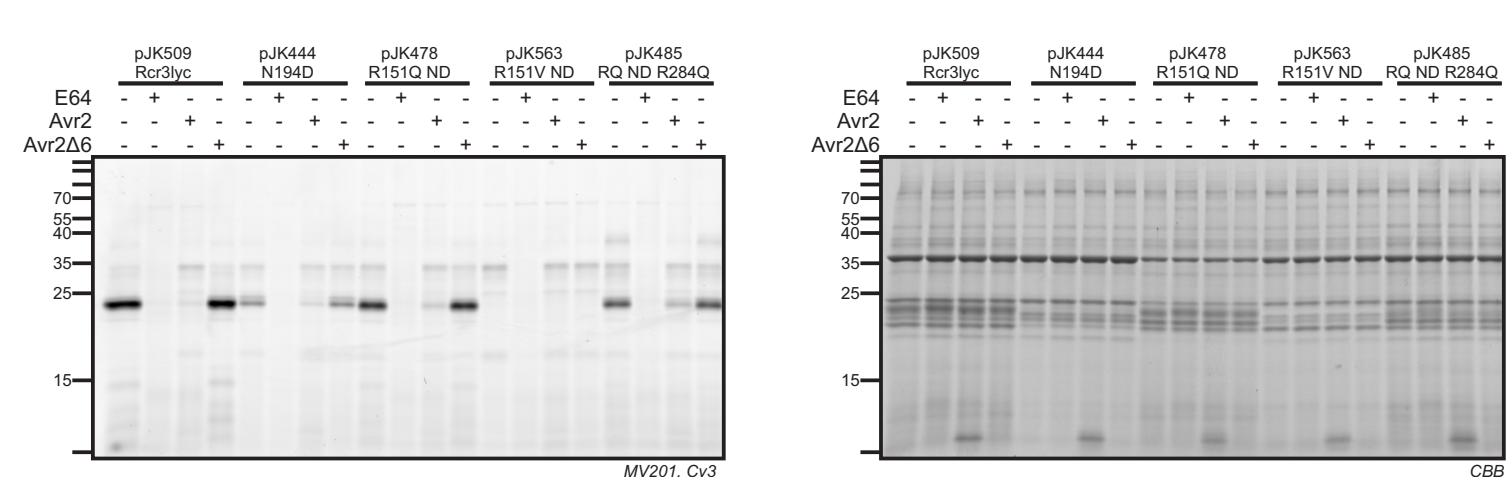

Figure 6d

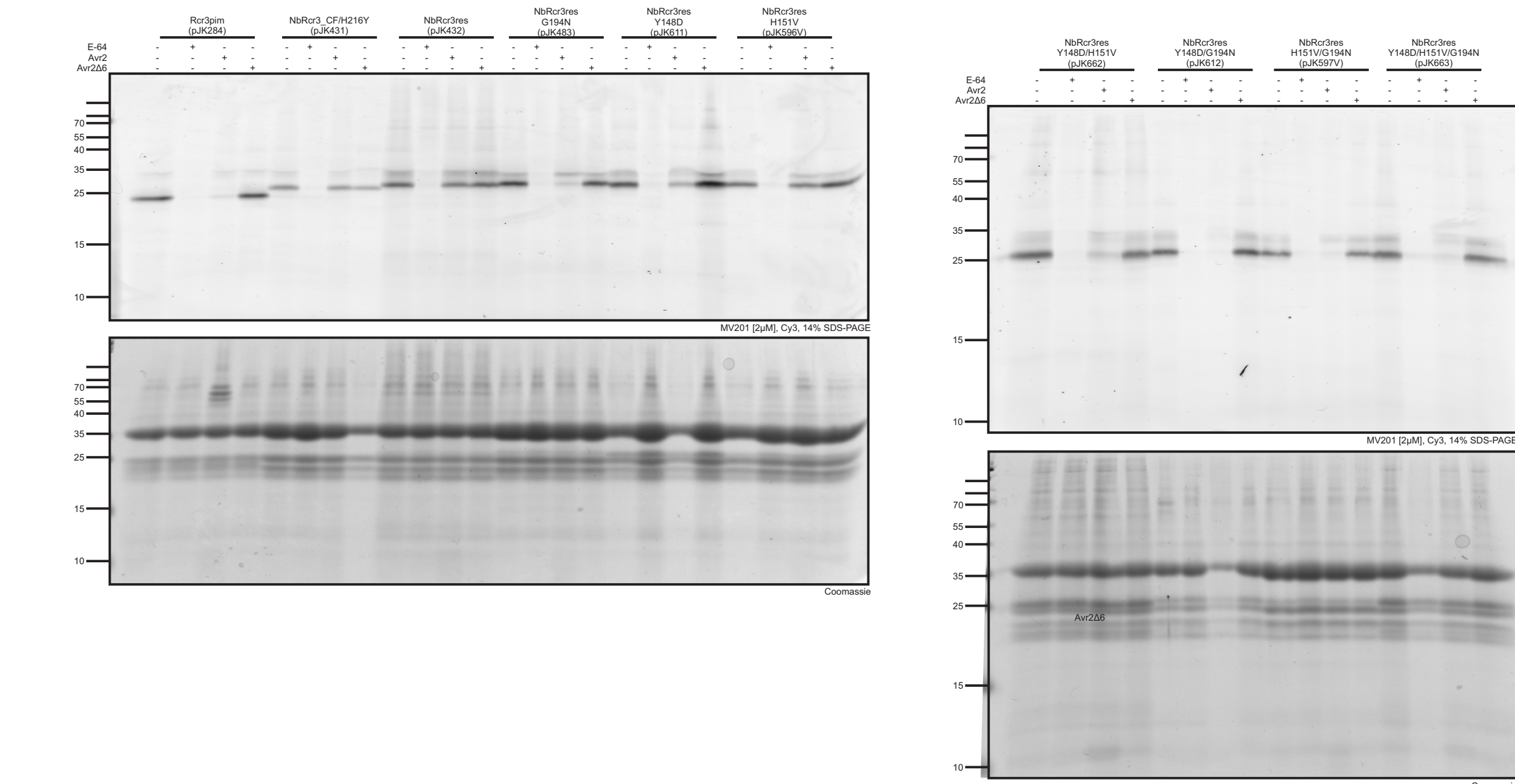

Figure S6a

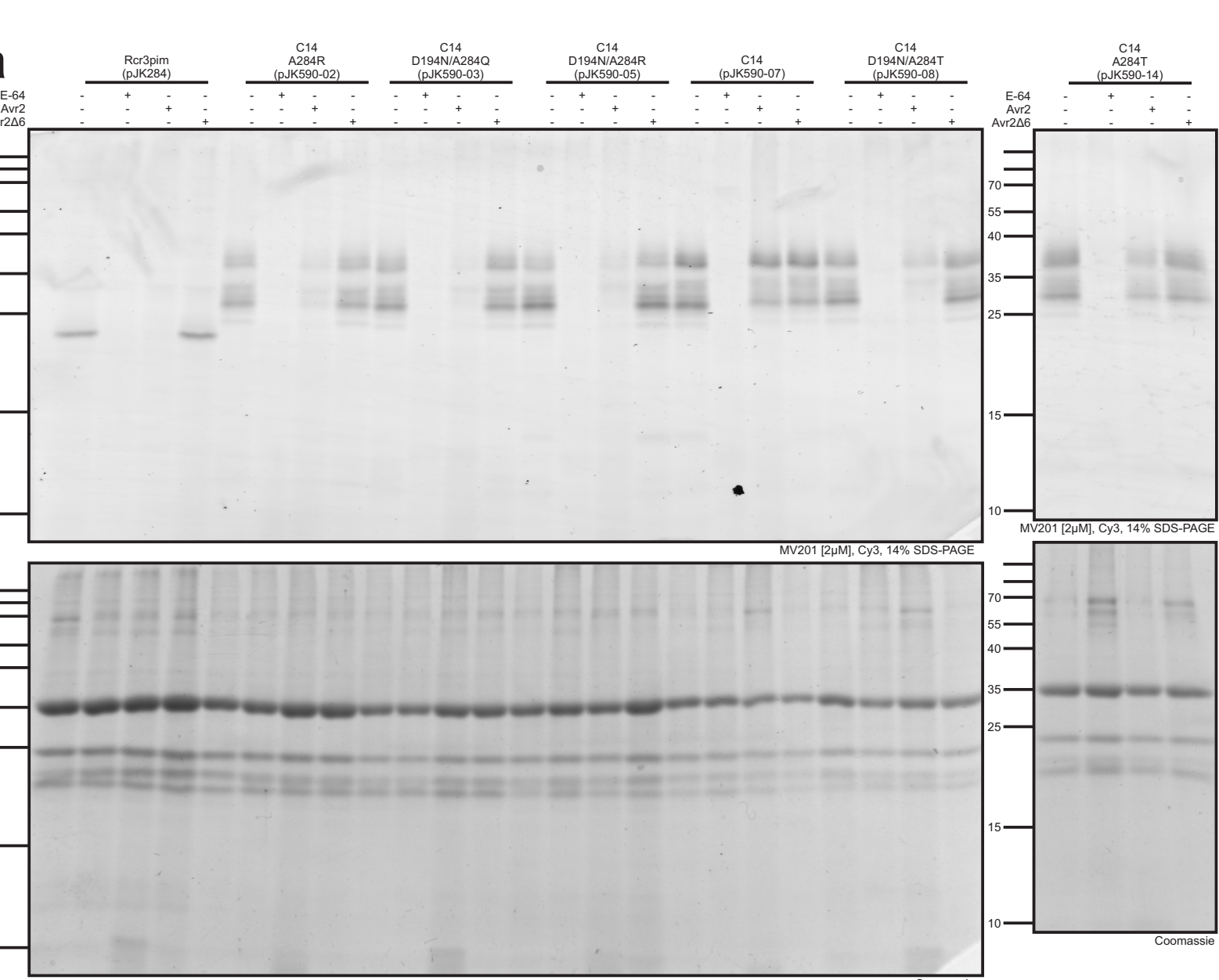

Figure S6b

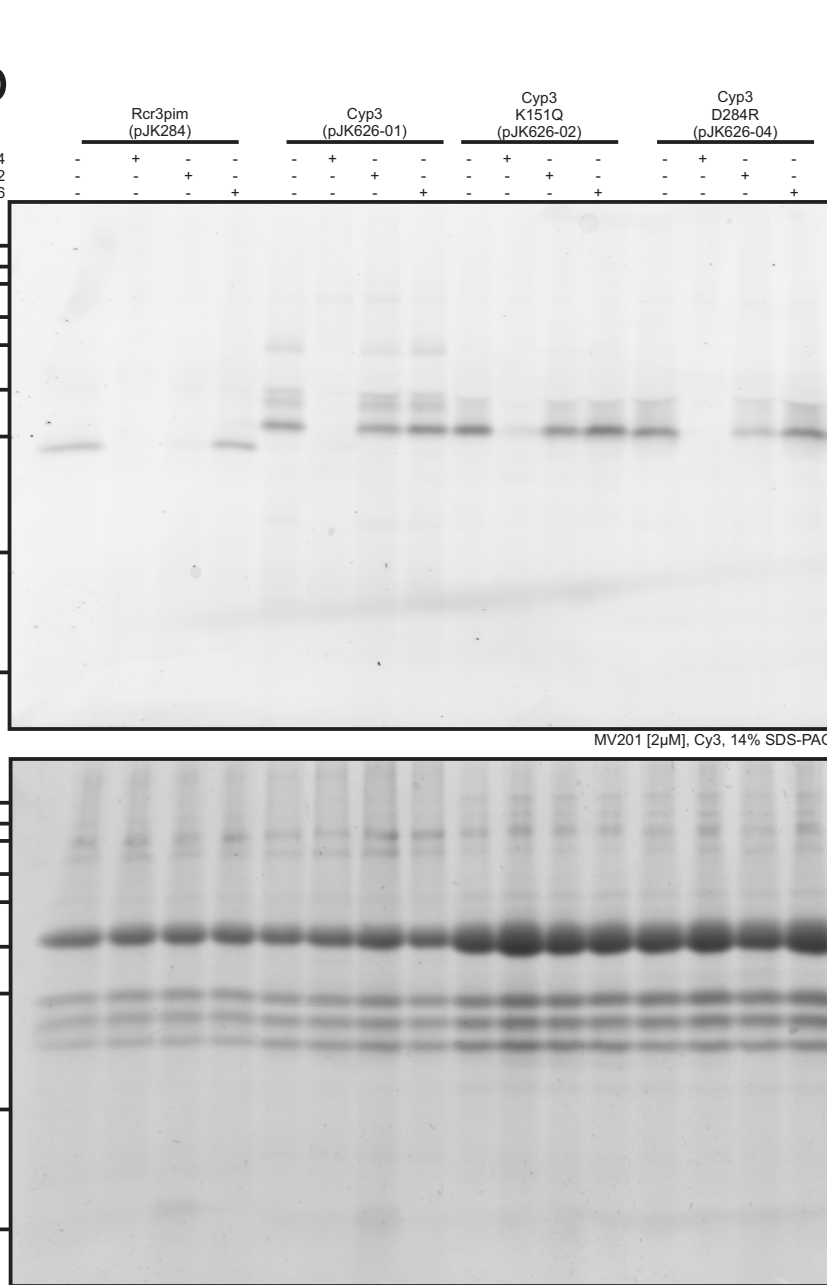

Supplement: Supplementary file 8 — Source Data [file 41467_2020_18069_MOESM8_ESM.zip › Source figures.pdf]
